# Apoptosis-Associated Gene Expression Profiling Is One New Prognosis Risk Predictor of Human Rectal Cancer

**DOI:** 10.1155/2022/4596810

**Published:** 2022-04-23

**Authors:** Qiansan Zhu, Xian Wu, Lili Ma, Haibo Xue

**Affiliations:** ^1^Department of Surgery, Wenzhou Hospital of Traditional Chinese Medicine Affiliated to Zhejiang Chinese Medicine University, Wenzhou, 325000 Zhejiang Province, China; ^2^Department of Rheumatology, The First Affiliated Hospital of Wenzhou Medical University, Wenzhou, 325000 Zhejiang Province, China; ^3^Department of Gastroenterology, The First Affiliated Hospital of Wenzhou Medical University, Wenzhou, 325000 Zhejiang Province, China

## Abstract

**Background:**

Prior research has revealed the predictive significance of a series of genetic markers in the prognosis of rectal cancer (RC), but the roles of apoptosis-associated genes in RC are rarely studied.

**Methods:**

The RNA-seq data as well as clinical data about patients with rectum adenocarcinoma (READ) were downloaded from The Cancer Genome Atlas (TCGA) and the Genotype-Tissue Expression (GTEx) project. Additionally, 87 apoptosis-associated genes were downloaded and acquired from Kyoto Encyclopedia of Genes and Genomes (KEGG) database. Comprehensive bioinformatics analysis was carried out for deep exploration of the expression and prognostic significance of these genes. Least absolute shrinkage and selection operator (LASSO) and multivariate Cox regression analysis was performed for the establishment of a risk scoring equation for the prognosis model and construction of a survival prognosis model. ROC curves were drawn for evaluating the accuracy of the model. A real-time quantitative PCR assay was conducted for quantification of apoptosis-associated proteins related to prognosis.

**Results:**

Eight genes were identified as hub genes associated with the prognosis of PFS. A risk model of prognosis prediction based on four gene signatures (CYCS, IKBKB, NFKB1, and TRADD) was constructed. According to further analysis of this model, the high-risk group experienced worse overall survival than the other. The prognosis model demonstrated a favorable predictive ability, with areas under the receiver operating characteristic curves (AUC) of 0.720, 0.641, and 0.677 in forecasting the 1-, 2-, and 3-year prognosis, respectively. In addition, CYCS and NFKB1 presented low expression, while IKBKB and TRADD presented high expression in TCGA and clinical tumor samples.

**Conclusions:**

A four-gene signature risk model for prognosis forecasting of RC has been constructed, which possesses favorable predictive ability, which offers ideas and breakthrough points to the apoptosis-associated development of RC.

## 1. Introduction

According to the World Cancer Report 2020, colorectal cancer (CRC) ranks second in frequently seen cancers among both men and women [1]. Appropriately 30%-35% of CRC patients are rectal cancer (RC) patients, most of which are rectal adenocarcinoma (READ) [2]. It is the tumor from the dentate line to the border of the rectum and sigmoid colon, which can be easily confirmed through sigmoid colonoscopy and digital rectal examination, and mainly afflicts individuals > 45 years old [3, 4]. RC can be treated by surgery, radiotherapy, chemotherapy, molecular targeted therapy, etc. [5–8]. Clinically, surgical resection is still the primary means for it. However, many patients with cancer miss the optimal surgery timing because of the lack of notable symptoms in the early phase, so the treatment outcome of advanced RC is still unsatisfactory [9]. CRC is classified into colon cancer and RC [10, 11]. Despite their various common characteristics, the two have some crucial differences, including the high postoperative local recurrence rate of appropriately 15%-45% in RC [12]. Through years of efforts, the number of oncogenes and tumor suppressors and markers of RC has been clarified [13, 14]. However, the risk model for prognosis prediction of READ requires further research. Besides, there are abundant studies on colon cancer than rectal cancer which motivated us to perform this study to construct a prediction model of prognosis for rectal cancer patients. The innovation of this study is to construct a new and promising risk model for predicting the prognosis of rectal cancer. Accordingly, it is of crucial significance to find and establish a prognosis model of READ for developing strong diagnosis and therapy methods and evaluating the prognosis.

Apoptosis, or programmed cell death, also takes a pivotal part in the development and maturation of normal tissues. It maintains the homeostasis *in vivo* by controlling the immune system, as the main cellular mechanism that eliminates DNA-damaged cells and maintains the homeostasis of tissues for mammals [15–17]. There exist two primary ways to activate apoptosis, the external way and the internal way. Tumors can escape apoptosis in many means. For instance, the increase in BCL-2 protein and deletion of BAX/BAK suppress the apoptosis and thus trigger tumor [18], and suppression of caspase function also hinders apoptosis function [19]. In addition, *in vivo* and *in vitro* assays have verified the roles of many chemopreventive drugs in inducing transformed cells' apoptosis [20, 21].

Zhang et al. [22] have confirmed some metabolic genes associated with RC prognosis. Huang et al. [23] have identified a risk model based on 10 M6A gene markers for prognosis prediction of RC patients. However, the application of apoptosis-associated genes in forecasting the prognosis of RC is yet to be reported. Accordingly, this study analyzed the expression and associations of 87 apoptosis-associated genes in RC and used LASSO regression and Cox regression analyses to screen out prognosis-associated gene markers. Finally, a prognostic risk prediction model composed of four gene signatures was constructed, which had high accuracy.

## 2. Methods

### 2.1. Data Source

RNA-seq as well as clinical data about READ cases were acquired from The Cancer Genome Atlas (TCGA, https://portal.gdc.cancer.gov/) and RNA-seq data of healthy tissues from the Genotype-Tissue Expression (GTEx, https://gtexportal.org/) project, removing batch effects from normalized data and corresponding to the corresponding clinical samples, removing duplicates and deleted samples from the downloaded data and cases with missing clinical outcomes. For the analysis of patients' clinical data, the data of those with unknown survival time and those with survival time of “0” were deleted. 87 apoptosis-associated genes were acquired from the Kyoto Encyclopedia of Genes and Genomes (KEGG) database.

### 2.2. Specimen Collection

Totally, 33 RC tissue specimens and 33 corresponding paracarcinoma tissue specimens were acquired from RC patients who underwent surgery in the Gastroenterology Department in the 1^st^ Affiliated Hospital of Wenzhou Medical University and the Large Intestine Surgery Department in Wenzhou Central Hospital between January 2019 and July 2020. The resected RC tissues and paracarcinoma tissues were immediately placed in liquid nitrogen for later analyses. All the postoperative specimens were confirmed by pathological examination, and the site that cancer tissues were taken was a cancer-rich site, and no obvious necrotic tissue was found in the specimens. Our study was performed after ratification by the hospital Medical Ethics Committee.

### 2.3. Gene Expression Analysis

The Limma software package of R software (version 4.0.3) was adopted for studying the differential expression of mRNA. The adjusted *P* value was analyzed based on TCGA or GTEx to correct the false positive result. Under the condition of |logFC|≧1 and adjusted *P* < 0.05, genes with aberrant expression were screened, and the heat map was drawn via R package ggplot2.

### 2.4. Protein-Protein Interaction (PPI) Network Construction

Genes with differential levels were evaluated using Metascape (https://metascape.org/gp/index.html#/main/step1), and PPI network was thus constructed. The MCODE plugin in Cytoscape software (version 3.8.2) was adopted for further exploration of gene interaction.

### 2.5. Kaplan-Meier Survival Analyses

Survival in R package was utilized for survival analyses. *P* value and hazard ratio (95% CI) in KM curves were acquired through both log-rank test and univariate Cox proportional hazard regression.

### 2.6. Gene Ontology (GO) Enrichment Analysis

According to GO analysis of genes based on DAVID (version 6.8, https://david.ncifcrf.gov/), *P* < 0.05 or FDR < 0.05 in the enrichment was significant in terms of statistics.

### 2.7. LASSO Model Construction

With the LASSO regression algorithm, characteristics were selected, and 10-fold cross-validation was carried out for parameter determination to acquire a suitable model. Then, the genes acquired by LASSO regression were treated with multivariate Cox regression, and the multivariate regression coefficient of every gene was calculated, on which a risk scoring equation was constructed. Patients were grouped into the high-/low-risk groups in the light of the median risk score. KM survival curves were used for analyzing and comparing the two groups' overall survival (OS), and time-associated receiver operator characteristic curve (ROC) was adopted for predictive value evaluation of gene markers.

### 2.8. Protein Expression Verification

Immunohistochemical staining maps about protein levels of gene signatures in CRC tissues and normal rectum tissues were acquired from the Human Protein Atlas (HPA).

### 2.9. Gene Set Enrichment Analysis (GSEA)

To observe the effect of gene expression on tumors, the samples were divided into two groups of high and low expression based on the median expression level of genes, and the enrichment of KEGG and HALLMARK pathways in the high- and low-expression group was analyzed using GSEA, respectively.

### 2.10. Real-Time Quantitative PCR (RT-qPCR)

Total RNA of tissues acquired via Trizol reagent (Invitrogen, Carlsbad, California, the States) were subjected to reverse transcription under the guidelines of PrimeScript RT Reagent Kit (Takara, Japan) to acquire cDNA that was subsequently treated by PCR under the guidelines of SYBR Premix Ex Taq II Kit (Takara, Japan). Shanghai Sangon Biotech Co., Ltd. completed the synthesis of specific primers adopted in qRT-PCR (Table 1: primer sequences). The calculation of relative expression was carried out using 2^-*ΔΔ*Ct^ (internal reference: GAPDH). The assay was conducted in triplicate, and the obtained results were averaged.

### 2.11. Statistical Analyses

SPSS 22.0 (IBM Corp., Armonk, NY, USA) was adopted for data analyses and GraphPad Prism 8.0 (GraphPad Software, La Jolla California USA) for visualization of data into figures. Measurement data were presented by (mean ± SD), and every assay was performed in triplicate. The independent-samples *t*-test was adopted for intergroup comparison of measurement data in normal distribution. Differentially expressed genes between normal tissues and tumor tissues were identified through the Wilcoxon test. *P* < 0.05 implies a notable difference.

## 3. Results

### 3.1. Expression and Interaction of Apoptosis-Associated Genes

**Figure 1:**
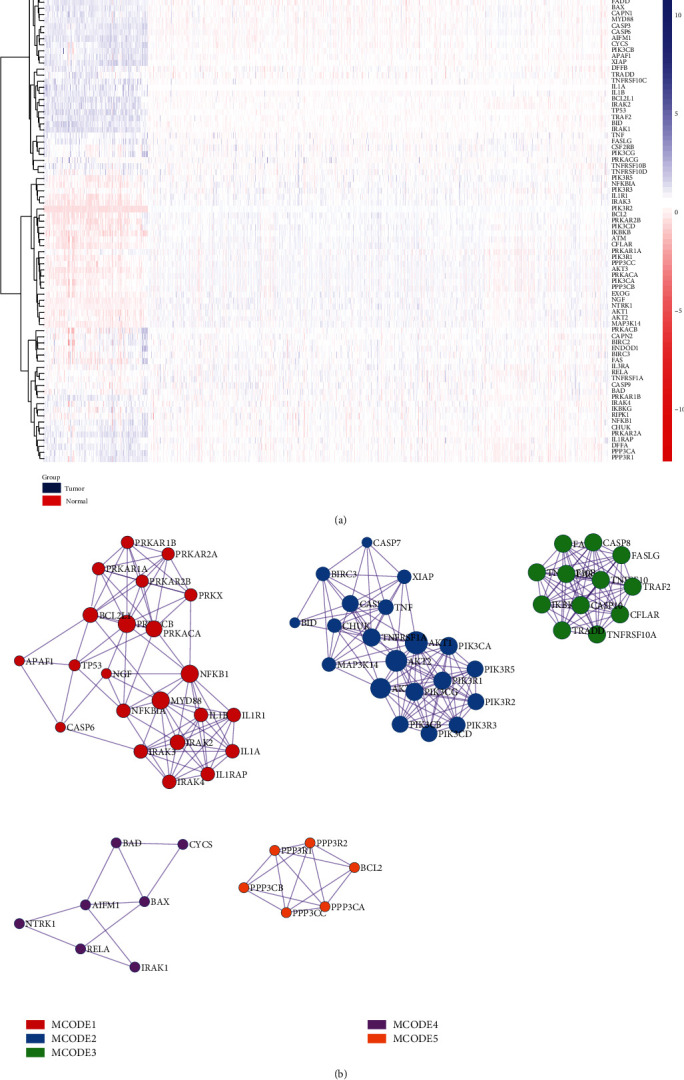
Expression and interaction of apoptosis-associated genes in READ. (a) The heat map displays the expression of apoptosis-associated genes in specimens from TCGA+GTEx. (b) PPI network displays the interaction of apoptosis-associated genes.

Based on observation on the expression of 87 apoptosis-associated genes in 165 RC specimens and 10 corresponding paracancerous specimens from TCGA and 799 normal tissue specimens from GTEx, 77 genes with differential levels were acquired via variation analysis (Figure 1(a): the heat map). For exploring the interaction of these apoptosis-associated genes, PPI was conducted, and the MCODE plugin in Cytoscape was adopted for the calculation of characteristics of each node in the network diagram and their visualization (Figure 1(b)). The functional enrichment of all MCODE modules is presented in Table 2. According to the results, 5 MCODEs were mainly enriched in IL-1 signaling pathway, apoptosis, c-FLIP regulation, and amyotrophic lateral sclerosis (ALS).

### 3.2. Progression-Free Survival (PFS) Analysis of 77 Genes

**Figure 2:**
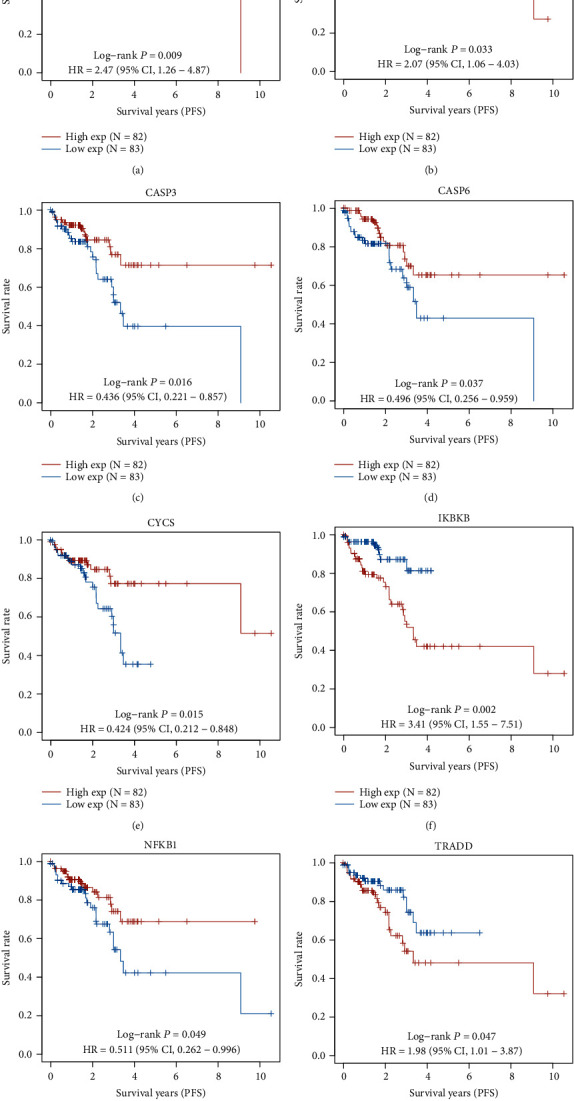
Progress-free survival analysis. (a) KM curves for PFS of AKT2. (b) KM curves for PFS of CAPN1. (c) KM curves for PFS of CASP3. (d) KM curves for PFS of CASP6. (e) KM curves for PFS of CYCS. (f) KM curves for PFS of IKBKB. (g) KM curves for PFS of NFKB1. (h) KM curves for PFS of TRADD.

According to the clinical information of 165 specimens, the PFS of 77 genes was analyzed, and 8 PFS-associated genes bound up with READ were acquired (Figures 2(a)–2(h): KM curves for PFS of them). All specimens were grouped into high-/low-expression groups based on gene level (median). According to the obtained results, high expression of AKT2, CAPN1, IKBKB, and TRADD and low expression of CASP3, CASP6, CYCS, and NFKB1 were all strongly bound up with short PFS in patients (all *P* < 0.05).

### 3.3. Establishment of a Risk Model for Prognosis Prediction Based on TCGA Cohort

**Figure 3:**
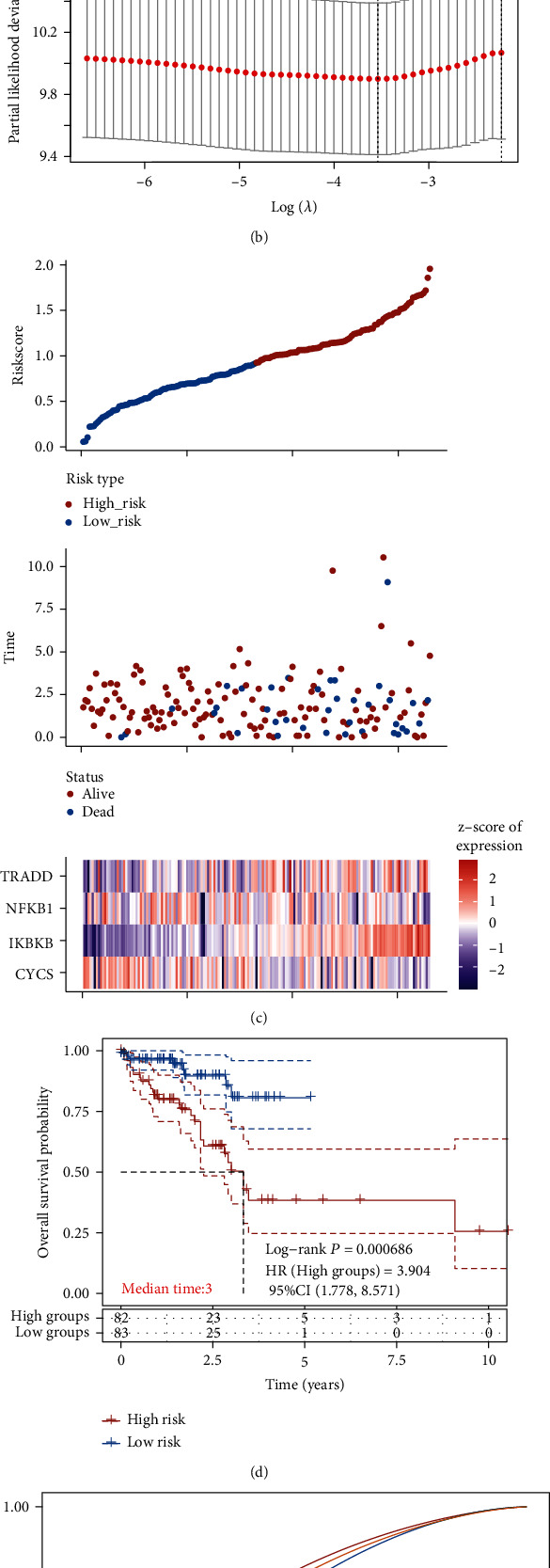
Construction of a risk model based on TCGA cohort. (a) LASSO regression analysis of 7 PFS-associated apoptosis genes. (b) Intersectional confirmation for parameter choice in LASSO regression analysis. (c) Survival time, risk score, state, and levels of 4 gene signatures. (d) KM curves of the high-/low-risk groups. (e) ROC curves for accuracy verification of LASSO model in prediction within 1, 2, and 3 years.

With the LASSO-Cox regression model, prognostic characteristics were constructed for analysis of gene levels. According to the minimum criterion (lambda.min = 0.0291), CYCS, IKBKB, NFKB1, and TRADD were selected for construction of a prediction model (Figures 3(a) and 3(b)) with four gene signatures. The prediction risk score was primarily composed of the following: risk score = (0.7211)∗IKBKB + (0.058)∗TRADD + (−0.1209)∗CYCS + (−0.2872)∗NFKB1.

According to the median value of risk scores, these specimens were grouped into the high-/low-risk groups. As the distribution of survival time showed in Figure 3(c), more patients died in the high-risk group than in the low-risk group, and there were more patients with shorter overall survival (OS) in the high-risk group than in the low-risk group. KM analysis revealed notably worse prognosis in the high-risk group than in the other (Figure 3(d)). Additionally, the sensitivity and specificity of this model for forecasting the OS of high-/low-risk cases were verified via ROC curves. The AUC of the risk model was 0.720, 0.641, and 0.677 in forecasting the 1-, 2-, and 3-year prognosis, respectively, demonstrating relatively high accuracy in forecasting the prognosis and survival of READ patients (Figure 3(e)).

### 3.4. Independent Predictive Significance of the Risk Model

We conducted multivariate and univariate Cox regression analyses for evaluating the feasibility of adopting the risk scores of four gene signatures as one independent factor for prognosis. According to the latter analysis, in TCGA cohort, risk score and TMN staging were independent factors for forecasting adverse survival (Table 3). According to the former analysis, risk score was one independent prognostic predictor after adjustment or other confounding factors (Table 4).

### 3.5. Expression of CYCS, IKBKB, NFKB1, and TRADD in TCGA-READ

**Figure 4:**
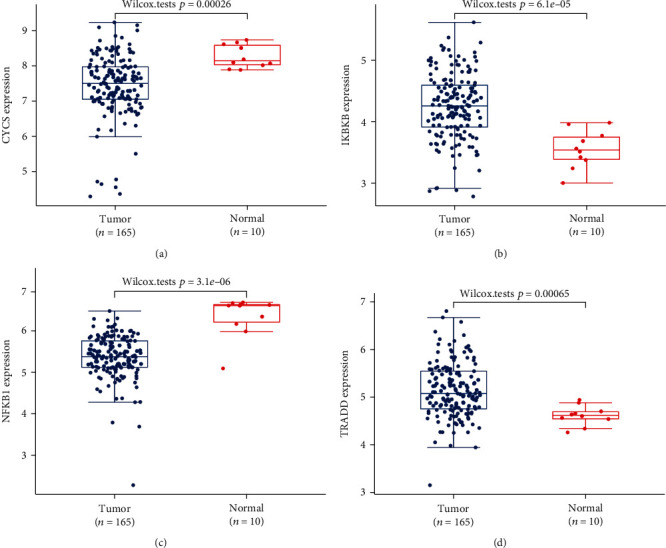
Expression of CYCS, IKBKB, NFKB1, and TRADD: (a) CYCS expression; (b) IKBKB expression; (c) NFKB1 expression; (d) TRADD expression.

According to expression analysis of CYCS, IKBKB, NFKB1, and TRADD in 165 RC tissue specimens and 10 corresponding paracarcinoma tissue specimens from TCGA, RC tissues showed downregulated CYCS and NFKB1 (Figures 4(a) and 4(c)) and upregulated IKBKB and TRADD (Figures 4(b) and 4(d)) in contrast to normal tissues.

### 3.6. Expression of CYCS, IKBKB, NFKB1, and TRADD in Clinical Specimens

**Figure 5:**
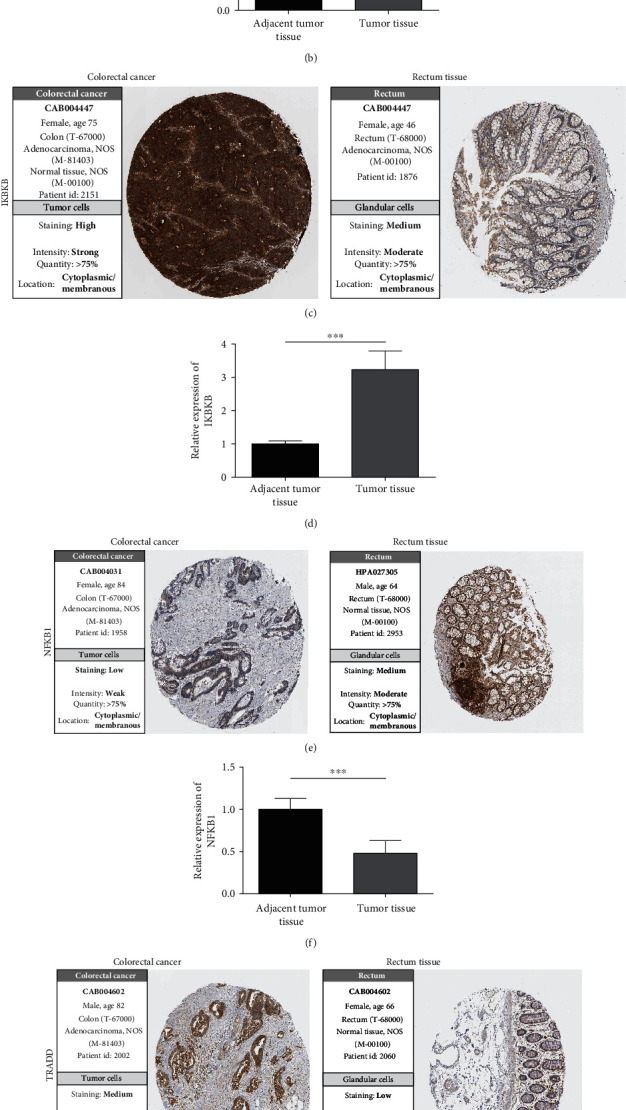
Protein expression verification of 4 genes and their expression in clinical specimens. (a) Expression verification of CYCS in cancer tissues and normal rectum tissues based on HPA. (b) CYCS in clinical specimens. (c) Expression verification of IKBKB in cancer tissues and normal rectum tissues based on HPA. (d) IKBKB in clinical specimens. (e) Expression verification of NFKB1 in cancer tissues and normal rectum tissues based on HPA. (f) NFKB1 in clinical specimens. (g) Expression verification of TRADD in cancer tissues and normal rectum tissues based on HPA. (h) TRADD in clinical specimens. ^∗∗∗^*P* < 0.001.

The HPA contained the immunohistochemical results of 4 genes in CRC tissues and normal rectum tissues. The results revealed expressed CYCS and NFKB1 in normal rectum tissues and their lower expression in CRC tissues (Figures 5(a) and 5(e)) and also revealed underexpressed IKBKB and TRADD in normal rectum tissues and their higher expression in CRC tissues (Figures 5(c) and 5(g)). In the collected clinical specimens, qPCR results revealed notably lower CYCS and NFKB1 expression (Figures 5(b) and 5(f)) and notably higher IKBKB and TRADD expression (Figures 5(d) and 5(h)) in cancer tissues than in healthy paracarcinoma ones (*P* < 0.05).

### 3.7. Gene Set Enrichment Analysis

**Figure 6:**
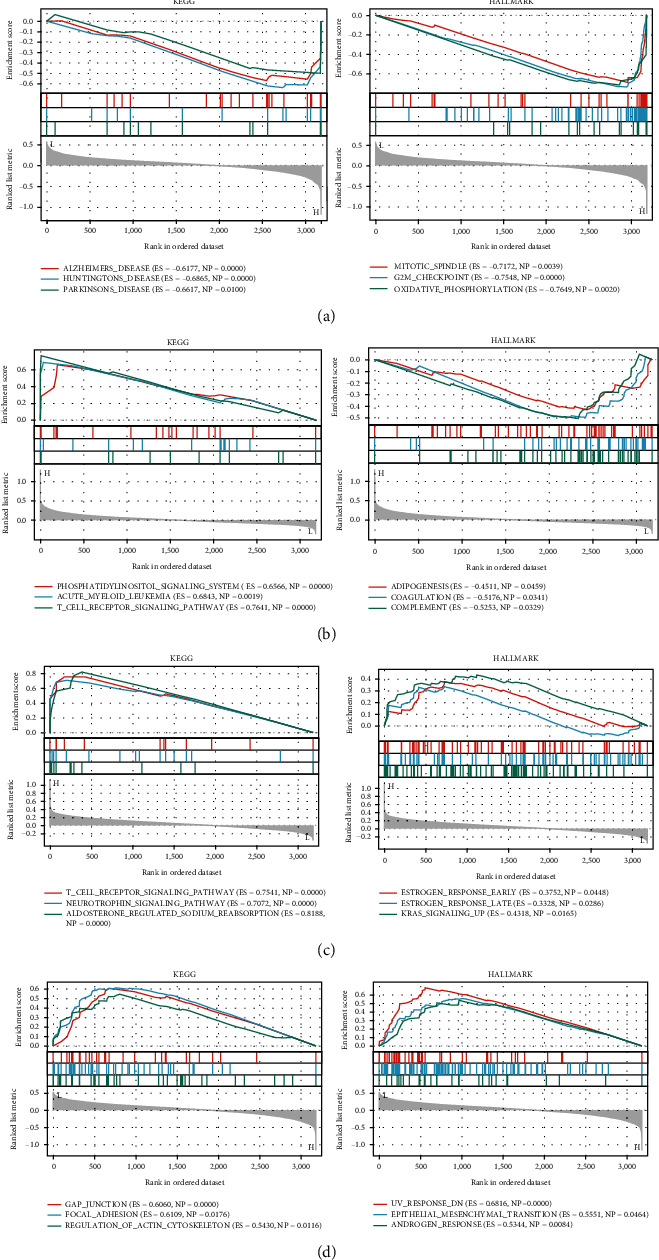
Gene set enrichment analysis of 4 genes in KEGG and HALLMARK datasets. (a) GSEA results of CYCS ranked in the top 3 for its correlation with signaling pathways in KEGG and HALLMARK database. (b) GSEA results of NFKB1 ranked in the top 3 for its correlation with signaling pathways in KEGG and HALLMARK database. (c) GSEA results of IKBKB ranked in the top 3 for its correlation with signaling pathways in KEGG and HALLMARK database. (d) GSEA results of TRADD ranked in the top 3 for its correlation with signaling pathways in KEGG and HALLMARK database.

To observe the effect of gene expression on tumors, we divided the rectal cancer samples into two groups with high and low expression according to the median expression levels of CYCS, NFKB1, IKBKB, and TRADD and analyzed the enrichment of signaling pathways in KEGG and HALLMARK in high- and low-expression groups by GSEA. The top 3 signaling pathways most significantly enriched in both databases have been listed. GSEA results verified CYCS was mainly enriched in Alzheimer diseases and mitotic spindle (Figure 6(a)). NFKB1 was mainly enriched in phosphatidylinositol signaling system and adipogenesis (Figure 6(b)). IKBKB was mainly enriched in T cell receptor signaling pathway and early estrogen response (Figure 6(c)). TRADD was mainly enriched in gap junction and UV response (Figure 6(d)).

## 4. Discussion

RC is one frequently seen cancer, with an unfavorable postoperative cure rate and OS and a high local recurrence rate. Over the past few years, searching for gene markers associated with RC prognosis and developing other prediction methods for prognosis have become a hot focus [13, 24]. Our study acquired the information of apoptosis-associated genes and corresponding data from TCGA-READ dataset. Through LASSO regression analysis, a risk model for forecasting RC prognosis was constructed, which delivered high accuracy in forecasting patients' prognosis in 1, 2, and 3 years (AUC: 0.720, 0.641, and 0.677), with four apoptosis-associated gene signatures. Subsequently, multivariate and univariate Cox regression analyses demonstrated that the risk scores given to the 4 gene signatures could serve as independent prognostic markers.

The risk model for prognosis prediction of RC based on 4 apoptosis-related genes was composed of CYCS, IKBKB, NFKB1, and TRADD. Few studies were conducted to construct prediction model for the prognosis of rectal cancer. Zuo et al. [25] identified a 6-gene signature predicting prognosis for colorectal cancer including rectal cancer. Thus, our prediction model of prognosis in rectal cancer is of significant. Whereafter, verification results by HPA database and qPCR of clinical tissue specimens revealed lowly expressed CYCS and NFKB in RC tissues and highly expressed IKBKB and TRADD in them.

CYCS, released by mitochondria, forms a large protein complex called apoptotic bodies by interacting with Apaf-1. The complex recruits and activates caspase-9 to initiate caspase cascade and apoptosis and thus supports the synthesis of ATP in mitochondria. CYCS is also bound up with cancers involving apoptosis and p53 tumor inhibition pathway [26]. Liu et al. [27] have revealed that the upregulation of CYCS triggers the cascade activation of caspase-9 and caspase-3 and then gives rise to apoptosis. However, in our study, CYCS presented low expression in RC cases, and the low expression was strongly bound up with the short PFS. Similar to prior research, patients with a low CYCS level show weaker apoptosis of cancer cells. As everyone knows, the IKK complex is composed of IKK*α*, IKK*γ*, and IKK*β* (also referred as IKBKB) [28]. The last one has been verified to be implicated in tumor growth through NF-*κ*B activation and phosphorylation-dependent suppression of tumor suppressor [29, 30]. It has been confirmed to have increased expression and/or abnormal activity in various kinds of human cancers including osteosarcoma [31]. Gong et al. [32] have discovered the association of IKBKB rs2272736 with the survival rate of gastric cancer. Our study analyzed the PFS time of TCGA specimens and revealed a shorter PFS time of patients with high IKBKB expression. NFKB1 is one subunit of NF-*κ*B [33]. It has been verified to be one pathway-specific tumor inhibitor, which can prevent hematological malignancies after cell damage mediated by alkylating agent (N-methyl-N-nitrosourea) [34]. Prior research has revealed that NFKB1 acts via p50 homodimer and is a suppressor of neutrophil-driven hepatocarcinoma [35]. The significance of NFKB1 function could be observed in models of mouse: Nfkb1−/− mouse showed intensified inflammatory response and susceptibility to DNA injury in some forms and finally suffered cancer and quick aging phenotype [36]. However, in our study, NFKB1 presented low expression in RC cases, and the low expression was also strongly bound up short PFS. The results also indirectly reflect that downregulated NFKB1 could not act as a tumor suppressor gene, which induces RC development. TRADD is one crucial TNF signal transduction medium mediated by TNFR1. Its mechanism of action is primarily responsible for recruiting other effector proteins, thus activating MAPK, NF-*κ*B, and other signal pathways including cell death [37, 38]. On the one hand, TRADD takes a pivotal part in TNF-*α*-induced proinflammatory reaction by interacting with TNFR1 [39]. On the other hand, TNF-*α* also initiates apoptosis and necrosis by recruiting FADD protein [40]. Based on our study results, IKBKB, NFKB1, and TRADD are all bound up with the NF-*κ*B signaling pathway, which offers ideas to the further study of RC.

Based on the above research results, we constructed a model for the prediction of RC prognosis based on 4 apoptosis-associated genes and further revealed the feasibility of using risk score based on this model as one independent predictor of RC patients' prognosis. The results provide a potential novel target for RC therapy and also a novel target and idea for targeted therapy of tumors. The study also has some limitations. Genes in tumors are studied less, and their mechanism of action needs deeper exploration. Besides, the clinical tissue samples we collected for this study do not include the follow-up survival information which cannot be used for the validation of the risk model. Whether the conclusions already reached can be verified equally *in vivo* or *in vitro* needs to be proved by more follow-up basic biological experiments. In addition, due to the rare public RNA-seq datasets on rectal cancer, it is urgent to collect more clinical rectal cancer sample to construct a transcriptome library.

## Figures and Tables

**Table 1:** Primer sequences.

Genes	Primer sequences (5′-3′)
CYCS	Forward: CTTTGGGCGGAAGACAGGTC
	Reverse: TTATTGGCGGCTGTGTAAGAG

IKBKB	Forward: GTCTTTGCACATCATTCGTGGG
	Reverse: GTGCCGAAGCTCCAGTAGTC

NFKB1	Forward: GGTGCGGCTCATGTTTACAG
	Reverse: GATGGCGTCTGATACCACGG

TRADD	Forward: GCTGTTTGAGTTGCATCCTAGC
	Reverse: CCGCACTTCAGATTTCGCA

GAPDH	Forward: ACAACTTTGGTATCGTGGAAGG
	Reverse: GCCATCACGCCACAGTTTC

**Table 2:** GO enrichment analysis of each MCODE.

MCODE	GO	Description	log_10_(*P*)
MCODE_1	WP195	IL-1 signaling pathway	-21.8
MCODE_1	WP4496	Signal transduction through IL1R	-21.2
MCODE_1	R-HSA-9020702	Interleukin-1 signaling	-19.1
MCODE_2	ko04210	Apoptosis	-40.4
MCODE_2	hsa04210	Apoptosis	-39.6
MCODE_2	ko04668	TNF signaling pathway	-36
MCODE_3	R-HSA-3371378	Regulation under c-FLIP	-35.4
MCODE_3	R-HSA-69416	Procaspase-8 dimerization	-35.4
MCODE_3	R-HSA-5218900	Suppressed CASP8 activity	-35.4
MCODE_4	ko04210	Apoptosis	-13.2
MCODE_4	hsa04210	Apoptosis	-13
MCODE_4	M144	PID ceramide pathway	-13
MCODE_5	ko05014	Amyotrophic lateral sclerosis (ALS)	-16.8
MCODE_5	hsa05014	ALS	-16.5
MCODE_5	WP3414	Initiation of transcription and translation elongation at the HIV-1 LTR	-13.9

**Table 3:** Univariate Cox regression analysis.

	*B*	SE	Wald	*P* value	HR (95% CI)
Age	0.003	0.016	0.029	0.865	1.003 (0.972, 1.034)
Gender	-0.340	0.349	0.950	0.330	0.712 (0.360, 1.410)
TNM stage	0.830	0.196	17.935	0.000	2.293 (1.562, 3.366)
Risk score	1.302	0.412	10.007	0.002	3.677 (1.641, 8.239)

**Table 4:** Multivariable Cox regression analysis.

	*B*	SE	Wald	*P* value	HR (95% CI)
Age	-0.004	0.016	0.073	0.787	0.996 (0.964, 1.028)
Gender	-0.560	0.378	2.192	0.139	0.571 (0.272, 1.199)
TNM stage	0.691	0.206	11.284	0.001	1.996 (1.334, 2.987)
Risk score	1.126	1.126	5.838	0.016	3.084 (1.237, 7.688)

## Data Availability

Bioinformatics data from the TCGA and GEO public databases were used to support this study. These previous datasets have been attributed where relevant in the text (https://portal.gdc.cancer.gov/) (https://gtexportal.org/).

## References

[1] Sung H., Ferlay J., Siegel R. L. (2021). Global cancer statistics 2020: GLOBOCAN estimates of incidence and mortality worldwide for 36 cancers in 185 countries. *CA: a Cancer Journal for Clinicians*.

[2] Machackova T., Prochazka V., Kala Z., Slaby O. (2019). Translational potential of microRNAs for preoperative staging and prediction of chemoradiotherapy response in rectal cancer. *Cancers*.

[3] Wilkinson N. (2020). Management of rectal cancer. *The Surgical Clinics of North America*.

[4] Shulman K., Musallam S., Epelbaum R., Haim N., Ben-Yosef R., Kaidar-Person O. (2020). The older adults with rectal cancer—does age matter?: a single-center experience. *American Journal of Clinical Oncology*.

[5] Komen N., Dewint P., Van den Broeck S., Pauli S., de Schepper H. (2019). Rectal cancer surgery: what’s in a name. *Acta Gastro-Enterologica Belgica*.

[6] Glimelius B. (2020). Adjuvant chemotherapy in rectal cancer: state of the art and future perspectives. *Current Opinion in Oncology*.

[7] Myint A. S., Gérard J. P. (2020). Role of radiotherapy in the treatment of rectal cancer in older patients. *European Journal of Surgical Oncology*.

[8] Clifford R. E., Govindarajah N., Bowden D. (2020). Targeting acid ceramidase to improve the radiosensitivity of rectal cancer. *Cell*.

[9] Chen C.-H., Hsieh M.-C., Hsiao P.-K., Lin E.-K., Lu Y.-J., Wu S.-Y. (2017). A critical reappraisal for the value of tumor size as a prognostic variable in rectal adenocarcinoma. *Journal of Cancer*.

[10] Salibasic M., Pusina S., Bicakcic E. (2019). Colorectal cancer surgical treatment, our experience. *Medical Archives*.

[11] Iqbal A., George T. J. (2017). Randomized clinical trials in colon and rectal cancer. *Surgical Oncology Clinics*.

[12] Tamas K., Walenkamp A., De Vries E. (2015). Rectal and colon cancer: not just a different anatomic site. *Cancer Treatment Reviews*.

[13] Dayde D., Tanaka I., Jain R., Tai M. C., Taguchi A. (2017). Predictive and prognostic molecular biomarkers for response to neoadjuvant chemoradiation in rectal cancer. *International Journal of Molecular Sciences*.

[14] Peng J., Lin J., Qiu M. (2017). Oncogene mutation profile predicts tumor regression and survival in locally advanced rectal cancer patients treated with preoperative chemoradiotherapy and radical surgery. *Tumor Biology*.

[15] Xu X., Lai Y., Hua Z.-C. (2019). Apoptosis and apoptotic body: disease message and therapeutic target potentials. *Bioscience Reports*.

[16] Pistritto G., Trisciuoglio D., Ceci C., Garufi A., D'Orazi G. (2016). Apoptosis as anticancer mechanism: function and dysfunction of its modulators and targeted therapeutic strategies. *Aging (Albany NY)*.

[17] Carneiro B. A., El-Deiry W. S. (2020). Targeting apoptosis in cancer therapy. *Nature Reviews Clinical Oncology*.

[18] Gaumer S., Guenal I., Brun S., Theodore L., Mignotte B. (2000). Bcl-2 and Bax mammalian regulators of apoptosis are functional in ∗∗_Drosophila_∗∗. *Cell Death & Differentiation*.

[19] Shi Y. (2002). Mechanisms of caspase activation and inhibition during apoptosis. *Molecular Cell*.

[20] Mohammad R. M., Muqbil I., Lowe L. (2015). Broad targeting of resistance to apoptosis in cancer. *Seminars in Cancer Biology*.

[21] Fresco P., Borges F., Marques M., Diniz C. (2010). The anticancer properties of dietary polyphenols and its relation with apoptosis. *Current Pharmaceutical Design*.

[22] Zhang Z. Y., Yao Q. Z., Liu H. Y. (2020). Metabolic reprogramming-associated genes predict overall survival for rectal cancer. *Journal of Cellular and Molecular Medicine*.

[23] Huang W., Li G., Wang Z. (2021). A ten-N6-methyladenosine (m6A)-modified gene signature based on a risk score system predicts patient prognosis in rectum adenocarcinoma. *Frontiers in Oncology*.

[24] Zhang G., Ma W., Dong H. (2020). Based on histogram analysis: ADC_aqp_ derived from ultra-high b-value DWI could be a non-invasive specific biomarker for rectal cancer prognosis. *Scientific Reports*.

[25] Zuo S., Dai G., Ren X. (2019). Identification of a 6-gene signature predicting prognosis for colorectal cancer. *Cancer Cell International*.

[26] Bordé E. C., Ouzegdouh Y., Ledgerwood E. C., Morison I. M. (2011). Congenital thrombocytopenia and cytochrome C mutation: a matter of birth and death. *Seminars in Thrombosis and Hemostasis*.

[27] Liu Z.-T., Xiong L., Liu Z.-P., Miao X.-Y., Lin L.-W., Wen Y. (2014). In vivo and in vitro evaluation of the cytotoxic effects of Photosan-loaded hollow silica nanoparticles on liver cancer. *Nanoscale Research Letters*.

[28] Zong S., Jiao Y., Liu X. (2021). FKBP4 integrates FKBP4/Hsp90/IKK with FKBP4/Hsp70/RelA complex to promote lung adenocarcinoma progression via IKK/NF-*κ*B signaling. *Cell Death & Disease*.

[29] Yang J., Splittgerber R., Yull F. E. (2010). Conditional ablation of Ikkb inhibits melanoma tumor development in mice. *The Journal of Clinical Investigation*.

[30] Hu M. C.-T., Lee D.-F., Xia W. (2004). I*κ*B kinase promotes tumorigenesis through inhibition of forkhead FOXO3a. *Cell*.

[31] Pan F., Zhang J., Tang B., Jing L., Qiu B., Zha Z. (2020). The novel circ_0028171/miR-218-5p/IKBKB axis promotes osteosarcoma cancer progression. *Cancer Cell International*.

[32] Gong Y., Zhao W., Jia Q. (2020). IKBKB rs2272736 is associated with gastric cancer survival. *Pharmacogenomics and Personalized Medicine*.

[33] Best K. T., Lee F. K., Knapp E., Awad H. A., Loiselle A. E. (2019). Deletion of NFKB1 enhances canonical NF-*κ*B signaling and increases macrophage and myofibroblast content during tendon healing. *Scientific Reports*.

[34] Voce D. J., Schmitt A. M., Uppal A. (2015). *Nfkb1* is a haploinsufficient DNA damage-specific tumor suppressor. *Oncogene*.

[35] Wilson C., Jurk D., Fullard N. (2015). *NF κB1* is a suppressor of neutrophil-driven hepatocellular carcinoma. *Nature Communications*.

[36] Concetti J., Wilson C. L. (2018). NFKB1 and cancer: friend or foe?. *Cell*.

[37] Kim J.-Y., Lee J.-Y., Kim D.-G., Koo G.-B., Yu J.-W., Kim Y.-S. (2011). TRADD is critical for resistance to TRAIL-induced cell death through NF-*κ*B activation. *FEBS Letters*.

[38] Malinin N. L., Boldin M. P., Kovalenko A. V., Wallach D. (1997). MAP3K-related kinase involved in NF-_K_B induction by TNF, CD95 and IL-1. *Nature*.

[39] Yu S., Xie J., Xiang Y. (2019). Downregulation of TNF-*α*/TNF-R1 signals by AT-lipoxin A4 may be a significant mechanism of attenuation in SAP-associated lung injury. *Mediators of Inflammation*.

[40] Xu Z.-y., Zheng M.-x., Zhang L. (2017). Dynamic expression of death receptor adapter proteins tradd and fadd in *Eimeria tenella* -induced host cell apoptosis. *Poultry Science*.

